# Comparative effectiveness of acupuncture in sham-controlled trials for knee osteoarthritis: A systematic review and network meta-analysis

**DOI:** 10.3389/fmed.2022.1061878

**Published:** 2023-01-09

**Authors:** Boram Lee, Tae-Hun Kim, Stephen Birch, Terje Alraek, Hye Won Lee, Arya Nielsen, L. Susan Wieland, Myeong Soo Lee

**Affiliations:** ^1^KM Science Research Division, Korea Institute of Oriental Medicine, Daejeon, Republic of Korea; ^2^Korean Medicine Clinical Trial Center, Korean Medicine Hospital, Kyung Hee University, Seoul, Republic of Korea; ^3^School of Health Sciences, Kristiania University College, Oslo, Norway; ^4^Department of Community Medicine, Faculty of Health Sciences, National Research Center in Complementary and Alternative Medicine, Tromsø, Norway; ^5^KM Convergence Research Division, Korea Institute of Oriental Medicine, Daejeon, Republic of Korea; ^6^Department of Family Medicine and Community Health, Icahn School of Medicine at Mount Sinai, New York, NY, United States; ^7^Center for Integrative Medicine, University of Maryland School of Medicine, Baltimore, MD, United States

**Keywords:** acupuncture, knee osteoarthritis, systematic review, network meta-analysis, comparative effectiveness, acupuncture therapy, placebo

## Abstract

**Objectives:**

Although many trials have assessed the effect of acupuncture on knee osteoarthritis (KOA), its efficacy remains controversial. Sham acupuncture techniques are regarded as representative control interventions in acupuncture trials and sometimes incorporate the use of sham devices (base units) to support a non-penetrating needle. To achieve successful blinding, these trials also use acupuncture base units in the verum acupuncture group. Base units are not used in real-world clinical settings. We aimed to assess the effect sizes of verum and sham acupuncture for KOA in sham-controlled trials with or without base units.

**Methods:**

A total of 10 electronic databases for randomized controlled trials (RCTs) comparing the efficacy of verum manual acupuncture and sham acupuncture for the treatment of KOA were searched for articles published before April 12, 2022. The primary outcome was pain intensity, and the secondary outcomes included physical function. The first assessment after the end of treatment was chosen for analysis. Effect sizes are reported as standardized mean differences (SMDs) with 95% confidence intervals (95% CIs). The risk of bias was assessed using the Cochrane risk of bias tool, and publication bias was evaluated using a funnel plot and Egger’s test. The quality of evidence for estimates was evaluated using the Grading of Recommendations, Assessment, Development, and Evaluations (GRADE) approach.

**Results:**

Fifteen RCTs were included. There was generally a low risk of bias except for the difficulty in blinding acupuncture therapists (performance bias). Compared to verum acupuncture in sham-controlled trials using base units, verum acupuncture in sham-controlled trials without base units was more effective for improving pain (SMD −0.56, 95% CI −1.09 to −0.03) and function (SMD −0.73, 95% CI −1.36 to −0.10) in KOA. The quality of evidence for network estimates was moderate to low due to the risk of bias and imprecision.

**Conclusion:**

These findings suggest that verum acupuncture in different types of sham-controlled trials has different effect sizes for KOA. Because base units are not used in clinical settings, the results of verum acupuncture in sham-controlled trials with base units need to be interpreted carefully.

**Systematic review registration:**

https://www.researchregistry.com/browse-the-registry#registryofsystematicreviewsmeta-analyses/registryofsystematicreviewsmeta-analysesdetails/6269f962606c5e001fd8790c/, identifier reviewregistry1351.

## 1. Introduction

Acupuncture, which most commonly involves the insertion of fine needles into specific acupuncture points, is a non-pharmacologic treatment that has long been used for the treatment of knee osteoarthritis (KOA) (1–3). Although many trials have assessed the use of acupuncture for KOA, its efficacy remains controversial. Therefore, recent guidelines do not provide consistent results regarding the recommendation of acupuncture for KOA treatment (4–7).

One of the greatest issues raised in acupuncture efficacy trials is whether a sham control is an appropriate control intervention since all types of sham acupuncture techniques stimulate the skin and are therefore not physiologically inert (8–10). Sham acupuncture controls can be classified according to whether a sham control device is used. When sham-controlled trials use a device or base unit attached to the skin, this device must also be used in the verum acupuncture group to maintain participant blinding (11, 12). However, because the base unit reduces the practitioner’s ability to manipulate the needle, the unit may hinder efforts to elicit “de qi” in the verum arm, which in turn may reduce the effectiveness of verum acupuncture. The presence of a base unit is not seen in clinical practice, raising the possibility that the effects of verum acupuncture in sham-controlled trials using base units may not reflect the effectiveness of acupuncture in the real-world clinical setting (13). According to our recent study, verum acupuncture in sham-controlled trials using devices was significantly less effective for hot flashes in menopausal women than verum acupuncture in trials using shallow needling as a control (14). A previous study suggested evidence of the difference in effect sizes between two verum acupuncture types for the non-pain condition, hot flushes (14). It is necessary to assess whether this phenomenon is always consistent regardless of the patient’s condition and outcomes. In this sense, we hypothesized that verum acupuncture would have different effects on KOA in sham-controlled trials depending on whether base units were used.

Network meta-analysis (NMA) is a useful methodology that enables the comparative effectiveness of various interventions by synthesizing both direct and indirect evidence. Therefore, even without direct clinical trials, the comparative effect can be estimated and ranked based on indirect evidence, thereby reducing the cost burden of direct clinical trials and providing relevant evidence. Various systematic reviews synthesizing the clinical evidence of acupuncture in the treatment of KOA have been published (3, 15–17). However, most systematic reviews synthesized only direct evidence of acupuncture treatment through a pairwise meta-analysis (3, 15). There were also reviews that synthesized both direct and indirect evidence of acupuncture through an NMA (16, 17). However, one study evaluated the comparative effectiveness of acupuncture and other physical treatments on KOA (16), and another study evaluated the comparative effectiveness of various acupuncture techniques on KOA through NMAs (17). In addition, to the best of our knowledge, we could not find any study comparing the effect size of verum acupuncture for KOA in sham-controlled trials with or without base units. Therefore, we aimed to analyze the comparative direct and indirect evidence for their effect estimates using an NMA approach.

## 2. Methods

The protocol was registered in the Research Registry (reviewregistry1351). We reported this study in accordance with the Preferred Reporting Items for Systematic Reviews and Meta-Analyses extension statement incorporating NMAs (18).

### 2.1. Eligibility criteria

We included studies that met the following eligibility criteria.

(1) Study design: prospective randomized clinical trials (RCTs) without limitations on publication date or language.
(2) Participants: adult patients diagnosed with KOA without limitations on sex, race, or nationality.
(3) Interventions: As treatment interventions, verum manual acupuncture in sham-controlled trials with [AT (device)] or without [AT (not)] a sham acupuncture device was included. Studies in which acupuncture was used in conjunction with other stimuli, such as electroacupuncture and laser acupuncture, were excluded. As control interventions, sham acupuncture with [Sham AT (device)] or without [Sham AT (not)] sham acupuncture devices, such as the Park Sham needle and Streitberger needle, was included (11, 12). For Sham AT (not), various sham acupuncture treatment methods, including shallow needling at non-acupuncture points, were included. Studies comparing verum acupuncture and a waitlist control (WL) were also included. In these studies, verum acupuncture did not use base units; therefore, it was analyzed as AT (not). For the NMA, WL was selected as a reference comparator. Studies comparing acupuncture vs. sham acupuncture in addition to standard treatments such as therapeutic exercise in both groups were also included (Table 1).
(4) Outcomes: The primary outcome was post-treatment pain intensity measured by the Western Ontario and McMaster Universities Osteoarthritis Index (WOMAC) pain subscale, 0–100 mm Visual Analog Scale (VAS), or other validated scales. The secondary outcome included post-treatment physical function and stiffness measured by WOMAC function and stiffness subscales or other validated scales. For the units of analysis in the outcome assessment, we considered the first assessment after the end of treatment. When our outcome of interest was evaluated with various scales in the included study (for example, pain was evaluated with WOMAC and VAS), WOMAC was adopted as a priority in the analysis. If duplicates of the same study were published in more than one journal, we only included the most comprehensive report. Studies published only as abstracts and/or studies in which data for the outcomes of interest could not be obtained even after contacting the author of the paper were excluded.

**TABLE 1:** Eligibility intervention criteria of the review.

Interventions	Types
Treatment interventions	Manual acupuncture – AT (device): Verum acupuncture in sham device-controlled trials – AT (not): Verum acupuncture in sham-controlled trials without a sham device or in waitlist-controlled trials
Control interventions	– Sham AT (device): Sham acupuncture using sham acupuncture devices (such as the Park Sham needle and Streitberger needle) – Sham AT (not): Non-device sham acupuncture (such as shallow needling at non-acupuncture points) – WL: Waitlist control (reference comparator)

### 2.2. Data sources and search strategy

The following 10 databases were searched by one researcher (BL): 4 English databases (Medline, EMBASE, the Cochrane Central Register of Controlled Trials, and the Allied and Complementary Medicine Database), 4 Korean databases (the Oriental Medicine Advanced Searching Integrated System, the Korean Studies Information Service System, the Korean Medical Database, and ScienceON), 1 Chinese database (the China National Knowledge Infrastructure), and 1 Japanese database (CiNii) from their inception to April 12, 2022. The reference lists of relevant systematic reviews and included studies as well as the International Clinical Trials Registry Platform were searched to identify not only the articles published in peer-reviewed journals but also gray literature such as conference proceedings. There were no language restrictions imposed. Detailed search strategies are described in Supplementary Data Sheet 1 (Supplement 1).

### 2.3. Study selection and data extraction

The study selection was conducted using EndNote 20 (Clarivate Analytics, Philadelphia, PA, USA). After removing duplicate papers found in each database, eligible articles were initially selected by review of the title and abstract. After this, the full texts of any potentially eligible studies were retrieved and examined in detail to determine final inclusion.

The following information was extracted from the included studies using a pilot-tested form: study characteristics; details about the participants; treatment and control interventions; and outcomes, treatment duration, and adverse events. If data were insufficient or missing, the authors of the included studies were contacted *via* e-mail to request additional information.

The study selection and data extraction process was conducted by two researchers (BL and MSL) independently, and any disagreements were resolved through discussions.

### 2.4. Risk of bias assessment

The Cochrane risk of bias tool was used to assess the risk of bias of the individual studies (19). The tool evaluates the risk of selection bias (random sequence generation and allocation concealment), performance bias (blinding of participants and personnel), detection bias (blinding of outcome assessment), attrition bias (incomplete outcome data), reporting bias (selective reporting), and other biases. In case of other biases, the statistical and clinical homogeneity of baseline characteristics of participants, including mean age, sex, and disease severity, between the treatment and control groups was tested. Each item was rated as “low,” “unclear,” or “high” risk of bias. Two researchers (BL and MSL) independently assessed the items, and discrepancies between them were resolved through discussions.

### 2.5. Data analysis and synthesis

Descriptive analyses of the main characteristics were performed for all included studies. Pairwise meta-analysis for direct comparisons was conducted for studies using the same types of treatment and control interventions using Review Manager version 5.4 software (Cochrane, London, UK). A random-effects model was used for pairwise meta-analysis due to evident clinical heterogeneity between the included studies on factors such as acupuncture points.

A random-effects NMA based on the frequentist model was conducted for our outcome of interest using network packages in Stata/MP software version 16 (StataCorp LLC, College Station, TX, USA). The five-node network map [AT (device) vs. AT (not) vs. Sham AT (device) vs. Sham AT (not) vs. WL] was presented for each outcome measure, with the node sizes and thickness of lines indicating the number of patients and trials, respectively. NMA was performed only when clinical similarity, transitivity, and consistency were tested and satisfied, and effect estimates were described through the network league tables and interval plots. The inconsistency was tested either at the global level of the whole network (design-by-treatment interaction model) or the local level of specific comparisons (node splitting method). If there were no connected loops in the network, NMA was not conducted because transitivity and consistency could not be examined. Because pain and physical function, our outcomes of interest, were reported as various scales in the included studies, the standard mean difference (SMD) with 95% confidence interval (CI) was used for effect estimates of both pairwise meta-analysis and NMA. We ranked the interventions based on their surface under the cumulative ranking curve (SUCRA) statistic to identify the best treatment. If sufficient studies (n ≥ 10) were included, the potential publication bias was assessed using a funnel plot and Egger’s test.

### 2.6. Quality of evidence

For each NMA outcome, the quality of the evidence was assessed for direct, indirect, and network estimates using the Grading of Recommendations, Assessment, Development, and Evaluations (GRADE) approach (20).

For the quality of direct evidence, the risk of bias, inconsistency, indirectness, and publication bias of the meta-analysis results were assessed. For the quality of indirect evidence, the lowest ratings of the two direct comparisons forming the most dominant first-order loop and intransitivity were considered. A higher rating of the quality of direct or indirect evidence and incoherence and imprecision of NMA results were considered for assessing the quality of the evidence *via* NMA. Each quality of evidence was rated as “high,” “moderate,” “low,” and “very low.”

## 3. Results

### 3.1. Study selection and study characteristics

**FIGURE 1:**
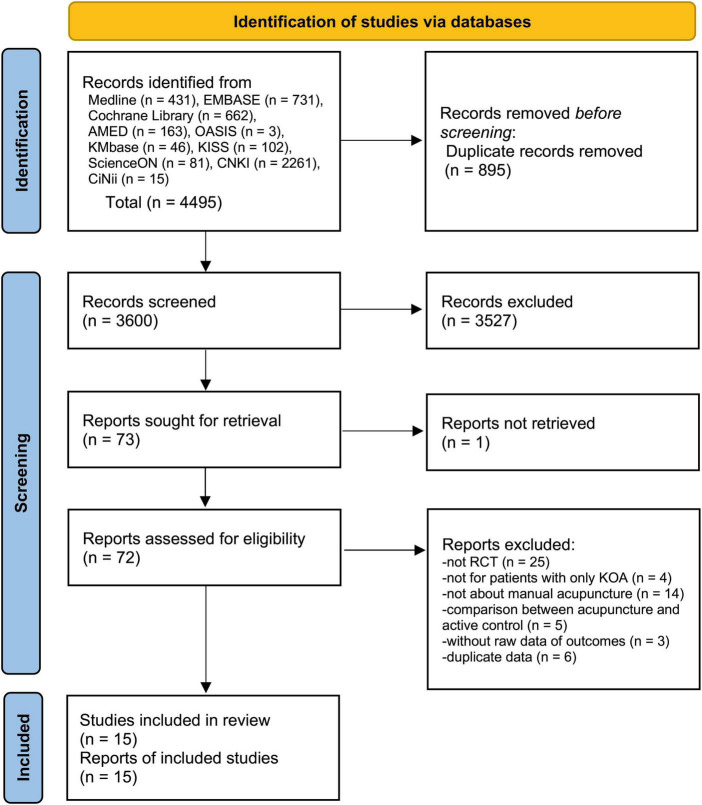
A PRISMA flow diagram of the literature screening and selection processes.

A total of 4,495 articles were searched through the electronic database. No additional studies were identified through the reference lists or trial registries. After removing 895 duplicate articles, the titles and abstracts of 3,600 articles were reviewed, and 73 were identified as potentially eligible. We retrieved full texts for 72 studies. After full text review, 57 articles were excluded for the following reasons: not RCTs (*n* = 25), not conducted for patients with KOA (*n* = 4), not evaluating manual acupuncture (*n* = 14), comparison between acupuncture and an active control (*n* = 5), no outcome data (*n* = 3), and duplicate data (*n* = 6) (Supplement 2 in Supplementary Data Sheet 1). Finally, a total of 15 studies (21–35) were included (Figure 1).

Among the included studies, there were 12 two-arm RCTs, including 3 studies comparing AT (device) vs. Sham AT (device) (23, 28, 29), 6 studies comparing AT (not) vs. Sham AT (not) (21, 31–35), and 3 studies comparing AT (not) vs. WL (25, 27, 30). There were 3 three-arm RCTs, including 1 study comparing AT (device) vs. Sham AT (device) vs. WL (26) and 2 studies comparing AT (not) vs. Sham AT (not) vs. WL (22, 24). Among them, five studies (24, 26, 28, 29, 33) administered standard treatment, such as exercise, in common to all groups. Although pain intensity was evaluated in all studies, only the median and interquartile results were presented in one study (33), and the mean and standard deviation values could not be confirmed even though contact with the corresponding author was attempted. Overall, 10 studies (21–26, 29–31, 34) used the WOMAC, 3 studies (27, 32, 35) used the VAS, and the remaining 1 study (28) used the Knee Society Score. There were 12 studies that evaluated physical function, of which 11 (21–26, 29–31, 33, 34) used the WOMAC and 1 (28) used the Knee Society Score. The network map of five nodes [AT (device) vs. AT (not) vs. Sham AT (device) vs. Sham AT (not) vs. WL] formed connected loops for pain and function outcomes (Supplements 3A, B in Supplementary Data Sheet 1). There were no significant inconsistencies between studies in the outcomes of pain or function at the global (pain, *p* = 0.4456; function, *p* = 0.6580) or local levels (Supplements 4, 5 in Supplementary Data Sheet 1). A total of 8 studies (21–25, 29, 33, 34) measured stiffness, and all of them used the WOMAC. In the included studies, a wide variety of acupuncture points, treatment frequencies, and numbers of treatments were used. The detailed study characteristics and detailed acupuncture treatment methods are described in Supplementary Data Sheet 1 (Supplements 6, 7).

### 3.2. Risk of bias assessment

Most of the studies were evaluated to have a low risk of selection bias by properly performing random sequence generation and allocation concealment. However, due to the nature of the research intervention, blinding of personnel could not be performed in all studies. Nine studies (21, 23, 28, 29, 31–35) comparing only acupuncture and sham acupuncture performed blinding of the participants regardless of whether a sham device was used. However, this was not possible in studies that included waitlist controls. In addition, 12 studies performed blinding of the outcome assessor, 2 (25, 35) did not, and 1 (30) did not mention this topic. All studies were evaluated to have a low risk of attrition and reporting bias (Supplement 8 in Supplementary Data Sheet 1).

### 3.3. Comparative effectiveness of verum acupuncture in sham-controlled trials

#### 3.3.1. Pain intensity

**FIGURE 2:**
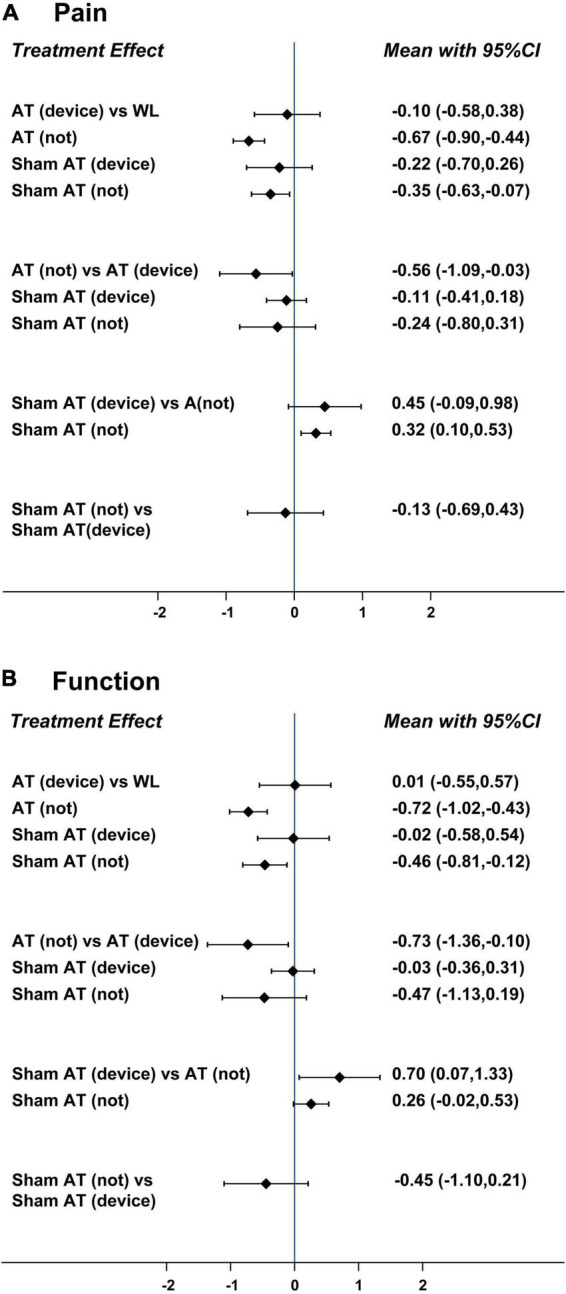
Interval plots of **(A)** pain intensity and **(B)** physical function. AT (device), verum acupuncture in sham device-controlled trials; AT (not), verum acupuncture in sham-controlled trials without a sham device; Sham AT (device), sham device control; Sham AT (not), non-device sham acupuncture; WL, waitlist.

From the NMA, the comparative effectiveness of AT (not) was significantly better than that of AT (device) (SMD −0.56, 95% CI −1.09 to −0.03). AT (not) was significantly effective compared to Sham AT (not) (SMD −0.32, 95% CI −0.53 to −0.10) and WL (SMD −0.67, 95% CI −0.90 to −0.44). However, there were no significant differences between AT (device) and Sham AT (device) (SMD 0.11, 95% CI −0.18 to 0.41) or between AT (device) and WL (SMD −0.10, 95% CI −0.58 to 0.38) (Figure 2A). The statistical significance of the pairwise meta-analysis and NMA results was consistent (Table 2; Supplement 9 in Supplementary Data Sheet 1). The funnel plot was visually symmetric, and there was no risk of publication bias in Egger’s test (*p* = 0.577) (Supplement 10 in Supplementary Data Sheet 1). The SUCRA plot suggested that AT (not) was ranked first, followed by Sham AT (not), Sham AT (device), AT (device) and WL in terms of the effect size for pain intensity (Supplement 11 in Supplementary Data Sheet 1).

**TABLE 2:** League table for pairwise meta-analysis (upper right) and network meta-analysis (lower left): pain intensity.

AT (device)	–	0.11 (−0.05, 0.28)	–	−0.12 (−0.38, 0.15)
**0.56 (0.03, 1.09)**	AT (not)	–	−**0.34 (**−**0.51,**−**0.17)**	−**0.63 (**−**0.95,**−**0.31)**
0.11 (−0.18, 0.41)	−0.45 (−0.98, 0.09)	Sham AT (device)	–	−0.21 (−0.47, 0.06)
0.24 (−0.31, 0.80)	−**0.32 (**−**0.53,**−**0.10)**	0.13 (−0.43, 0.69)	Sham AT (not)	−**0.50 (**−**0.73,**−**0.27)**
−0.10 (−0.58, 0.38)	−**0.67 (**−**0.90,**−**0.44)**	−0.22 (−0.70, 0.26)	−**0.35 (**−**0.63,**−**0.07)**	WL

The results are presented as the standard mean differences (95% confidence intervals). The comparison must be read from left to right. A standard mean difference less than zero indicates that treatment on the left is favored in both pairwise and network meta-analyses. A bold value indicates a significant difference between the groups. AT (device), verum acupuncture in sham device-controlled trials; AT (not), verum acupuncture in sham-controlled trials without a sham device; Sham AT (device), sham device control; Sham AT (not), non-device sham acupuncture; WL, waitlist.

#### 3.3.2. Physical function

From the NMA, the comparative effectiveness of AT (not) was significantly higher than those of AT (device) (SMD −0.73, 95% CI −1.36 to −0.10), Sham AT (device) (SMD −0.70, 95% CI −1.33 to −0.07), and WL (SMD −0.72, 95% CI −1.02 to −0.43). However, there was no significant difference between AT (device) and Sham AT (device) (SMD 0.03, 95% CI −0.31 to 0.36) or between AT (device) and WL (SMD 0.01, 95% CI −0.55 to 0.57) (Figure 2B). Although there was a statistically significant difference between AT (not) and Sham AT (not) in the pairwise meta-analysis (SMD −0.25, 95% CI −0.40 to −0.11), the statistical significance disappeared in the NMA (SMD −0.26, 95% CI −0.53 to 0.02). For other comparisons, the statistical significance of the pairwise meta-analysis and NMA results was consistent (Table 3; Supplement 12 in Supplementary Data Sheet 1). The funnel plot was visually symmetric, and there was no risk of publication bias in Egger’s test (*p* = 0.801) (Supplement 13 in Supplementary Data Sheet 1). The SUCRA plot suggested that AT (not) was ranked first, followed by Sham AT (not), Sham AT (device), AT (device) and WL in terms of effect size for physical function (Supplement 14 in Supplementary Data Sheet 1).

**TABLE 3:** League table for pairwise meta-analysis (upper right) and network meta-analysis (lower left): physical function.

AT (device)	–	0.06 (−0.10, 0.23)	–	0.00 (−0.26, 0.27)
**0.73 (0.10, 1.36)**	AT (not)	–	−**0.25 (**−**0.40,**−**0.11)**	−**0.71 (**−**1.13,**−**0.30)**
0.03 (−0.31, 0.36)	−**0.70 (**−**1.33,**−**0.07)**	Sham AT (device)	–	−0.01 (−0.28, 0.25)
0.47 (−0.19, 1.13)	−0.26 (−0.53, 0.02)	0.45 (−0.21, 1.10)	Sham AT (not)	−**0.58 (**−**1.05,**−**0.12)**
0.01 (−0.55, 0.57)	−**0.72 (**−**1.02,**−**0.43)**	−0.02 (−0.58, 0.54)	−**0.46 (**−**0.81,**−**0.12)**	WL

The results are presented as the standard mean differences (95% confidence intervals). The comparison must be read from left to right. A standard mean difference less than zero indicates that treatment on the left is favored in both pairwise and network meta-analyses. A bold value indicates a significant difference between the groups. AT (device), verum acupuncture in sham device-controlled trials; AT (not), verum acupuncture in sham-controlled trials without a sham device; Sham AT (device), sham device control; Sham AT (not), non-device sham acupuncture; WL, waitlist.

#### 3.3.3. Stiffness

Since there was no connected loop between sham-device controlled trials and sham-controlled trials without a sham device on the network map (Supplement 3C in Supplementary Data Sheet 1), it was not possible to review whether the assumptions of consistency and transitivity were satisfied. Therefore, only pairwise meta-analysis was conducted for the stiffness outcome. According to the pairwise meta-analysis, there were no significant differences between AT (device) and Sham AT (device) (2 studies, SMD 0.11, 95% CI −0.12 to 0.34, *I*^2^ = 0%). However, there were statistically significant differences between AT (not) and Sham AT (not) (4 studies, SMD −0.18, 95% CI −0.34 to −0.02, *I*^2^ = 37%), between AT (not) and WL (3 studies, SMD −0.70, 95% CI −1.02 to −0.39, *I*^2^ = 86%), and between Sham AT (not) and WL (2 studies, SMD −0.40, 95% CI −0.53 to −0.26, *I*^2^ = 0%).

### 3.4. Quality of evidence

The quality of evidence based on the GRADE approach for direct, indirect, and network estimates was analyzed. For outcomes of both pain intensity and physical function, the quality of evidence was moderate for both direct and indirect estimates, and the reasons for downgrading were related to the risk of bias of the studies included in the meta-analysis. The quality of evidence for network estimates was moderate to low, and it was downgraded for some comparisons due to the imprecision of the meta-analysis results (Supplement 15 in Supplementary Data Sheet 1).

## 4. Discussion

In this NMA, for the first time, we attempted to investigate the comparative effect estimates of what is essentially two types of verum acupuncture in sham-controlled trials with or without a sham device for KOA. As a result of a comprehensive database search, a total of 15 studies were included, and all of them had a generally low risk of bias except for the difficulty in blinding acupuncture therapists (performance bias), which occurs for an obvious reason. We found that AT (not) was significantly more effective in improving the pain and physical function of KOA than AT (device). The differences in the effects of the two verum acupuncture treatments on pain intensity and physical function had SMD values of 0.56 and 0.73, respectively, which could be considered medium differences (SMDs ranging from 0.5 to 0.8) (36). In studies comparing only verum and sham acupuncture, blinding of participants was successfully performed regardless of sham device use, which means that the difference in the effect of the two types of verum acupuncture is not due to the difference in blinding. These significant differences between the two verum acupuncture types are consistent with our previous study on the effect of verum acupuncture in different sham-controlled trials for hot flushes in menopausal women (14). The current study showed that AT (not) had statistically significant effects on improving pain intensity compared with Sham AT (not), and there was no difference between AT (device) and Sham AT (device). This is a difference from our previous study (14) in which there was no significant difference in the efficacy of verum and sham acupunctures regardless of the use of the sham device, and this may be due to differences in target diseases (pain and non-pain conditions) and the smaller sample sizes in the prior study. In addition, the current study showed its advantage of being more methodologically rigorous by comprehensively searching not only the core databases but also the local Korean, Chinese, and Japanese databases, by determining that there was no risk of potential publication bias through funnel plots and Egger’s test, and by assessing the quality of evidence for effect estimates. Interestingly, although it was reported that various types of sham controls can affect the effect size in acupuncture trials (37), in our study, only AT (not) and AT (device) had significant differences in pain and function outcomes, whereas Sham AT (not) and Sham AT (device) did not. That is, there was no difference in effect sizes between sham acupuncture types regardless of whether a device was used. These results suggest that the specific effects of verum acupuncture, but not the non-specific effects, are hampered by the use of the sham device.

The following limitations of our study should be considered. Although we performed NMA under the assumptions of similarity, transitivity, and consistency, the effect estimates between the two types of verum acupunctures were derived only from indirect evidence, and the quality of evidence for network estimates was moderate to low. Furthermore, there was unsolvable clinical heterogeneity in acupuncture points, treatment duration, and frequency among the included studies, although there were no significant inconsistencies between studies at the local and global levels. In addition, among the included studies, there were five studies in which standard treatment, such as physical therapy, was added to all intervention groups. To evaluate the potential impact of these studies, a subgroup analysis was attempted depending on whether standard treatment was performed in common for all groups. However, it was not possible because there were few included studies and it did not form a connected loop in the network map. Although standard treatment was performed for all groups in these studies, their potential clinical impact should be considered when interpreting the results.

Our findings suggest that different types of verum acupuncture might have non-identical effects for patients with pain conditions. This may be due to the base unit employed in sham device-controlled trials, which is required to hold a sham acupuncture device on the skin and is also used in the verum acupuncture group. Device use can minimize performance and detection bias by achieving participant blinding. However, it inhibits acupuncture manipulation by limiting stimulation depth, direction and needle perturbation and may not correspond to the effects of acupuncture stimulation seen in real-world acupuncture (13). In addition, the SUCRA plot suggested that AT (not) was ranked first, followed by Sham AT (not), Sham AT (device), AT (device) and WL in terms of effect size for both pain intensity and physical function. In particular, Sham AT (device) ranked prior to AT (device), although there was no statistical significance between them in the effect estimates of either pairwise meta-analysis or NMA. This may be due to the previously known physiological non-inertness of sham acupuncture (8–10) and the less effective results of acupuncture in the sham device-controlled trial confirmed in our study (possibly because of the use of base units in verum acupuncture). Therefore, the effect difference between verum and sham acupuncture for KOA in many previous studies may not have been significant, resulting in inconsistent recommendations of acupuncture for KOA treatment in recent guidelines (4–7). Consequently, there is a possibility that the effect of acupuncture for KOA may be underestimated. Based on the difference in effect derived from this NMA, it will be necessary to directly compare whether there is an effect difference between acupuncture in real-world clinical practice and that in a sham device-controlled trial. Additionally, pragmatic trials of acupuncture for KOA compared to usual care would help to clarify the potential benefit to patients in real-world clinical settings.

## 5. Conclusion

Verum acupuncture in sham-controlled trials with or without base devices appears to have different effect sizes for pain intensity and function in patients with KOA, and verum acupuncture in sham device-controlled trials may not be representative of the true effectiveness for KOA in clinical settings.

## Data availability statement

The original contributions presented in this study are included in the article/Supplementary material, further inquiries can be directed to the corresponding author.

## Author contributions

BL: methodology and writing—original draft preparation. MSL: conceptualization, methodology, funding acquisition, supervision, and writing—review and editing. T-HK: methodology and writing—review and editing. SB, TA, HWL, AN, and LSW: writing—review and editing. All authors contributed to the article and approved the submitted version.
