# Supplementary material for: Comparative effectiveness of acupuncture in sham-controlled trials for knee osteoarthritis: A systematic review and network meta-analysis
Source: Front Med (Lausanne). 2023 Jan 9;9:1061878. doi: 10.3389/fmed.2022.1061878 (PMC9868382; doi:10.3389/fmed.2022.1061878)
Supplement: Supplementary file 1 [file Data_Sheet_1.docx]

**Supplementary Online Contents**

**Supplement 1. Search strategy used in each database**

**Supplement 2. Excluded studies after full-text review**

**Supplement 3. Network map of (A) pain intensity, (B) physical function, and (C) stiffness**

**Supplement 4. Results of testing inconsistency at the local level through the node splitting method: Pain intensity**

**Supplement 5. Results of testing inconsistency at the local level through the node splitting method: Physical function**

**Supplement 6. Characteristics of included studies**

**Supplement 7. Details of acupuncture treatment method**

**Supplement 8. Risk of bias summary for all included studies**

**Supplement 9. Forest plot: Pain intensity**

**Supplement 10. Funnel plot: Pain intensity**

**Supplement 11. SUCRA plot: Pain intensity**

**Supplement 12. Forest plot: Physical function**

**Supplement 13. Funnel plot: Physical function**

**Supplement 14. SUCRA plot: Physical function**

**Supplement 15. Quality of evidence (GRADE)**

**Supplement 1. Search strategy used in each database**

**Medline via PubMed**

|  | Searches | Results |
| --- | --- | --- |
| #1 | "Osteoarthritis, Knee"[MH] OR ((osteoarthr*[TIAB] OR arthrosis[TIAB] OR "degenerative arthr*"[TIAB]) AND (Knee[MH] OR knee[TIAB] OR "Knee Joint"[MH])) OR KOA[TIAB] OR gonarthr*[TIAB] OR "knee pain"[TIAB] | 49,564 |
| #2 | Acupuncture[MH] OR “Acupuncture Therapy”[MH] OR “Acupuncture Points”[MH] OR acupunct*[TIAB] OR acupoint*[TIAB] OR “Dry Needling”[MH] OR “dry needling”[TIAB] OR "filiform needle"[TIAB] | 35,642 |
| #3 | “Randomized Controlled Trial”[PT] OR “Controlled Clinical Trial”[PT] OR randomized[TIAB] OR placebo[TIAB] OR “Clinical Trials as Topic”[Mesh: noexp] OR randomly[TIAB] OR trial[TI] | 1,531,875 |
| #4 | animals[MH] NOT humans[MH] | 4,990,325 |
| #5 | (#1 AND #2 AND #3) NOT #4 | **431** |

**EMBASE via Elsevier**

|  | Searches | Results |
| --- | --- | --- |
| #1 | 'knee osteoarthritis'/exp 'knee osteoarthritis':ab,ti OR 'knee arthritis'/exp OR 'knee arthritis':ab,ti OR ((osteoarthr*:ab,ti OR arthrosis:ab,ti OR 'degenerative arthr*':ab,ti) AND (knee/exp OR knee:ab,ti)) OR KOA:ab,ti OR gonarthr*:ab,ti OR 'knee pain'/exp OR 'knee pain':ab,ti | 73,771 |
| #2 | acupuncture/exp OR acupuncture*:ab,ti OR ‘acupuncture point’/exp OR ‘body meridian’/exp OR ‘body meridian’:ab,ti OR acupoint*:ab,ti OR 'dry needling'/exp OR 'dry needling':ab,ti OR ‘filiform needle’:ab,ti | 57,993 |
| #3 | 'crossover procedure':de OR 'double-blind procedure':de OR 'randomized controlled trial':de OR 'single-blind procedure':de OR (random* OR factorial* OR crossover* OR cross NEXT/1 over* OR placebo* OR doubl* NEAR/1 blind* OR singl* NEAR/1 blind* OR assign* OR allocat* OR volunteer*):de,ab,ti | 2,917,058 |
| #4 | #1 AND #2 AND #3 | **731** |

**CENTRAL**

|  | Searches | Results |
| --- | --- | --- |
| #1 | MeSH descriptor: [Osteoarthritis, Knee] explode all trees | 5,007 |
| #2 | (((osteoarthr* OR arthrosis OR "degenerative arthr*") AND Knee) OR KOA OR Gonarthr* OR "Knee pain"):ti,ab,kw | 15,302 |
| #3 | #1 OR #2 | 15,302 |
| #4 | MeSH descriptor: [Acupuncture] explode all trees | 161 |
| #5 | MeSH descriptor: [Acupuncture Therapy] explode all trees | 5,208 |
| #6 | MeSH descriptor: [Acupuncture Points] explode all trees | 2,222 |
| #7 | MeSH descriptor: [Dry Needling] explode all trees | 86 |
| #8 | (acupunct* OR acupoint* OR “dry needling” OR "filiform needle"):ti,ab,kw | 18,553 |
| #9 | #4 OR #5 OR #6 OR #7 OR #8 | 18,805 |
| #10 | (#3 AND #9) in Trials | **662** |

**AMED via EBSCO**

|  | Searches | Results |
| --- | --- | --- |
| #1 | "Osteoarthritis, Knee"[SU] OR KOA[TX] OR gonarthr*[TX] OR "knee pain"[TX] | 1,948 |
| #2 | (osteoarthr*[TX] OR arthrosis[TX] OR "degenerative arthr*"[TX]) AND (Knee[SU] OR knee[TX] OR "Knee Joint"[SU]) | 2,525 |
| #3 | Acupuncture[SU] OR “Acupuncture Therapy”[SU] OR “Acupuncture Points”[SU] OR acupunct*[TX] OR acupoint*[TX] OR “Dry Needling”[SU] OR “dry needling”[TX] OR "filiform needle"[TX] | 12,140 |
| #4 | (#1 OR #2) AND #3 | **163** |

**OASIS**

|  | Searches | Results |
| --- | --- | --- |
| #1 | (무릎\|슬관절\|슬통\|골관절염\|퇴행성관절염) (침) | **3** |

**KISS**

|  | Searches | Results |
| --- | --- | --- |
| #1 | 제목=(무릎\|슬관절\|슬통\|골관절염\|퇴행성관절염) AND 제목=(침) | **102** |

**KMbase**

|  | Searches | Results |
| --- | --- | --- |
| #1 | (((([TITLE=무릎] OR [TITLE=슬관절]) OR [TITLE=슬통]) OR [TITLE=골관절염]) OR [TITLE=퇴행성관절염]) | 2,729 |
| #2 | [TITLE=침] | 4,527 |
| #3 | #1 AND #2 | **46** |

**ScienceON**

|  | Searches | Results |
| --- | --- | --- |
| #1 | 논문명=(무릎\|슬관절\|슬통\|골관절염\|퇴행성관절염) (침) | **81** |

**CNKI**

|  | Searches | Results |
| --- | --- | --- |
| #1 | (SU=’膝关节炎’+'膝骨关节炎’+'膝骨性关节炎’+'膝关节骨关节炎’+'膝关节骨性关节炎’+'膝通’) and (SU=’针’) | **2,261** |

**CiNii**

|  | Searches | Results |
| --- | --- | --- |
| #1 | (膝関節炎 OR 膝骨関節炎 OR 膝変形性関節症 OR 膝の変形性関節症 OR 膝の痛み) AND 鍼 | **15** |

**Supplement 2. Excluded studies after full-text review**

**- Nonrandomized controlled trials: 25**

1. 江育清, 莫倩云, and 杨顺益, *退行性膝关节炎针灸治疗研究进展.* 中国民间疗法, 2002(12): p. 57-58.

2. 景传生, *中西医结合治疗骨性膝关节炎.* 深圳中西医结合杂志, 2000(03): p. 125-129.

3. 唐华伟, *温阳通经法治疗老年性膝关节炎78例临床观察.* 中医正骨, 2005(09): p. 69.

4. 杜昌华, *耳穴埋针治疗骨质增生性膝关节炎30例.* 浙江中医杂志, 1999(04): p. 166.

5. 廖仲围, *针刺治疗膝关节炎136例.* 中国民间疗法, 2001(10): p. 14.

6. 林雷 and 张华, *中西医结合治疗退行性膝关节炎.* 少年体育训练, 2008(01): p. 56-57.

7. 林耀庚, *针刺大巨穴治疗增生性膝关节炎40例.* 天津中医, 1999(05): p. 29-30.

8. 徐江文, *中西医结合治疗骨性膝关节炎.* 实用中西医结合临床, 2004(05): p. 56-57.

9. 王文欣 and 黄琳娜, *膝四针为主治疗增生性膝关节炎38例.* 中国针灸, 2000(11): p. 16.

10. 王淑琴, 姜丽, and 赵淑杰, *针刺为主治疗骨性膝关节炎49例.* 吉林医学, 2005(06): p. 610.

11. 宁玲 and 范庆花, *针灸治疗增生性膝关节炎365例.* 中国民间疗法, 2002(07): p. 10-11.

12. 曾焕芝, *中西医结合治疗马属动物变形性膝关节炎.* 当代畜牧, 2010(09): p. 21-22.

13. 曾焕芝, *中西结合治疗马属动物变形性膝关节炎.* 中兽医医药杂志, 2011. **30**(01): p. 53-54.

14. 彭易雨, *针刺膝眼穴为主治疗膝关节炎120例.* 实用中医药杂志, 2002(01): p. 30-31.

15. 刘长信. *退行性膝关节炎的中医综合疗法*. in *2012中国康复医学会实用康复论坛暨运动康复新理念培训班*. 2012. 中国河南安阳.

16. 劳力行. *针刺治疗膝关节炎疗效的临床试验*. in *第三届国际中医药工程学术会议*. 2006. 中国上海.

17. 张建平, *针灸温通法治疗退行性膝关节炎:针具不同疗效有差异.* 中国临床康复, 2002(16): p. 2464.

18. 张丁丁, *针灸疗法可减轻膝关节炎患者疼痛并改善关节功能.* 国外医学情报, 2005(04): p. 16-17.

19. 张洪军 and 郭明芳, *针刺为主治疗增生性膝关节炎200例.* 上海针灸杂志, 2002(03): p. 45.

20. Boylan, M., *Acupuncture with Voltaren is superior than Voltaren alone in knee osteoarthritis.* J Aust Tradit Med Soc, 2005. **11**(2).

21. Fargas-Babjak, A.M., B. Pomeranz, and P.J. Rooney, *Acupuncture-like stimulation with codetron for rehabilitation of patients with chronic pain syndrome and osteoarthritis.* Acupuncture & electro-therapeutics research, 1992. **17**(2): p. 95‐105.

22. Jiang, A., et al., *Clinical effect of acupuncture treatment in 109 cases of knee osteoarthritis.* Journal of Traditional Chinese Medicine, 2001. **21**(4): p. 282-5.

23. Liu, T. and C. Liu, *Acupuncture for treating osteoarthritis of the knee and the hip.* Arthritis and Rheumatism, 2006. **54**(11): p. 3375-3377.

24. Tebbutt, E., *Osteoarthritis of the knee in an elderly patient, treated with acupuncture.* J Acupunct Assoc Chart Physiotherapists, 2004. **2004**(1): p. 51-8.

25. Zhou, S.F. and C.C. Xue, *Acupuncture as an adjunct to exercise-based physiotherapy does not improve the pain of knee osteoarthritis.* Australian journal of acupuncture and chinese medicine, 2008. **3**(1): p. 53‐55.

**- Not only for patients with knee osteoarthritis: 4**

1. Christensen, B.V., et al., *Acupuncture treatment of severe knee osteoarthrosis. A long-term study.* Acta anaesthesiologica Scandinavica, 1992. **36**(6): p. 519‐525.

2. Christensen, B.V., et al., *Acupuncture treatment of knee arthrosis. A long-term study.* Ugeskrift for laeger, 1993. **155**(49): p. 4007‐4011.

3. Huang, W., et al., *Acupuncture for pain and sleep in knee osteoarthritis.* Journal of the American Geriatrics Society, 2010. **58**(6): p. 1218‐1220.

4. Molsberger, A., et al., *[Acupuncture treatment for the relief of gonarthrosis pain-a controlled clinical trial.].* Schmerz, 1994. **8**(1): p. 37-42.

**- Not about manual acupuncture: 14**

1. 徐杰, et al., *中西医结合治疗增生性膝关节炎34例.* 医学理论与实践, 2004(05): p. 549.

2. 鲍圣涌, 张少君, and 陈竞芬, *中西医结合治疗增生性膝关节炎的临床观察.* 湖北中医药大学学报, 2012. **14**(02): p. 52-53.

3. Berman, B.M., et al., *Effectiveness of acupuncture as adjunctive therapy in osteoarthritis of the knee: a randomized, controlled trial.* Annals of internal medicine, 2004. **141**(12): p. 901‐910.

4. Berman, B.M., et al., *A randomized trial of acupuncture as an adjunctive therapy in osteoarthritis of the knee.* Rheumatology (Oxford, England), 1999. **38**(4): p. 346‐354.

5. Jubb, R.W., et al., *A blinded randomised trial of acupuncture (manual and electroacupuncture) compared with a non-penetrating sham for the symptoms of osteoarthritis of the knee.* Acupuncture in medicine, 2008. **26**(2): p. 69‐78.

6, Mavrommatis, C.I., et al., *Acupuncture as an adjunctive therapy to pharmacological treatment in patients with chronic pain due to osteoarthritis of the knee: a 3-armed, randomized, placebo-controlled trial.* Pain, 2012. **153**(8): p. 1720‐1726.

7. Suarez-Almazor, M.E., et al., *A randomized controlled trial of acupuncture for osteoarthritis of the knee: Effects of patient-provider communication.* Arthritis Care and Research, 2010. **62**(9): p. 1229-1236.

8. Vas, J., C. Mendez, and E. Perea-Milla, *Acupuncture versus Streitberger needle in knee osteoarthritis -- an RCT.* Acupuncture medicine, 2006. **24**(Suppl): p. S15‐S24.

9. Vas, J., et al., *Acupuncture as a complementary therapy to the pharmacological treatment of osteoarthritis of the knee: randomised controlled trial.* BMJ (Clinical research ed.), 2004. **329**(7476): p. 1216.

10. White, A., et al., *Western medical acupuncture in a group setting for knee osteoarthritis: results of a pilot randomised controlled trial.* Pilot Feasibility Stud, 2016. **2**: p. 10.

11. Farazdaghi, M., et al., *Dry needling trigger points around knee and hip joints improves function in patients with mild to moderate knee osteoarthritis.* Journal of bodywork and movement therapies, 2021. **27**: p. 597‐604.

12. Romero, E.A.S., et al., *Is a Combination of Exercise and Dry Needling Effective for Knee OA?* Pain medicine (Malden, Mass.), 2020. **21**(2): p. 349‐363.

13. Sánchez-Romero, E.A., et al., *Effects of dry needling in an exercise program for older adults with knee osteoarthritis.* Medicine (United States), 2018. **97**(26).

14. Vervullens, S., et al., *The effect of one dry needling session on pain, central pain processing, muscle co-contraction and gait characteristics in patients with knee osteoarthritis: a randomized controlled trial.* Scand J Pain, 2022. **22**(2): p. 396-409.

**- Comparison between acupuncture and active control: 5**

1. 涂小华, *毫针傍刺治疗退行性膝关节炎.* 现代医药卫生, 2002(09): p. 801.

2. 李保军 and 杨巧凤, *针刺为主治疗增生性膝关节炎120例.* 上海针灸杂志, 2002(03): p. 31.

3. 徐菁 and 张大同, *阿是穴避痛埋针治疗老年退行性膝关节炎的疗效观察.* 中国中医药科技, 2016. **23**(03): p. 355-356.

4. 肖建墙. *关节腔内注射玻璃酸钠联合针灸治疗骨性膝关节炎的效果评价*. in *国际数字医学会数字中医药分会成立大会暨首届数字中医药学术交流会*. 2016. 中国广东珠海.

5. Zhang, J.-G., *Clinical effect observation of knee joint degenerative osteoarthropathy treated with acupuncture.* Journal of clinical acupuncture and moxibustion, 2003. **19**(11): p. 21.

**- No raw outcome data: 3**

1. Itoh, K., et al., *Trigger point acupuncture for treatment of knee osteoarthritis--a preliminary RCT for a pragmatic trial.* Acupuncture in medicine, 2008. **26**(1): p. 17‐26.

2. Karner, M., et al., *Objectifying specific and nonspecific effects of acupuncture: a double-blinded randomised trial in osteoarthritis of the knee.* Evidence-based complementary and alternative medicine, 2013. **2013**.

3. Spaeth, R.B., et al., *A longitudinal study of the reliability of acupuncture deqi sensations in knee osteoarthritis.* Evidence-based complementary and alternative medicine, 2013. **2013**.

**- Duplicate data: 6**

1. 古屋, 英. and 美. 直本, *Acupuncture for osteoarthritis of the knee.* 医道の日本 = The Japanese journal of acupuncture & manual therapies : 東洋医学・鍼灸マッサージの専門誌, 2012. **71**(10): p. 71-73.

2. Brinkhaus, B., et al., *Physician and treatment characteristics in a randomised multicentre trial of acupuncture in patients with osteoarthritis of the knee.* Complementary therapies in medicine, 2007. **15**(3): p. 180‐189.

3. Brinkhaus, B., et al., *Efficacy of acupuncture in patients with osteoarthritis of the knee. A randmized controlled trial.* Gynakologische Praxis, 2006. **30**(3): p. 539‐549.

4. Foster, N.E., et al., *The relationship between patient and practitioner expectations and preferences and clinical outcomes in a trial of exercise and acupuncture for knee osteoarthritis.* European journal of pain (London, England), 2010. **14**(4): p. 402‐409.

5. Manheimer, E., et al., *Acupuncture for knee osteoarthritis - a randomised trial using a novel sham.* Acupuncture in medicine, 2006. **24**: p. 7‐14.

6. Tu, J. F., et al., *Effect of acupuncture on knee injury and osteoarthritis outcome score in patients with knee osteoarthritis*. Zhongguo zhen jiu [Chinese acupuncture & moxibustion], 2021. **41**(1): p. 27‐30.

**Supplement 3. Network map of (A) pain intensity, (B) physical function, and (C) stiffness**

| (A) Pain |
| --- |
|  |
| (B) Function |
|  |
| (C) Stiffness |
|  |

AT(device), verum acupuncture in sham device-controlled trials; AT(not), verum acupuncture in sham-controlled trials without a sham device; Sham AT(device), sham device control; Sham AT(not), nondevice sham acupuncture; WL, waitlist.

Node sizes and thickness of lines indicate the number of patients and trials, respectively.

Supplement 4. Results of testing inconsistency at the local level through the node splitting method: Pain intensity

| Side | Direct comparison | | Indirect comparison | | Difference | | p-value |
| --- | --- | --- | --- | --- | --- | --- | --- |
|  | Coefficient | SE | Coefficient | SE | Coefficient | SE |  |
| AT(device) WL | 0.1139872 | 0.283118 | 0.0570346 | 0.614907 | 0.0569526 | 0.675879 | 0.933 |
| AT(device) Sham AT(device) | . | . | . | . | . | . | . |
| AT(not) WL | 0.6383054 | 0.1244647 | 1.018516 | 0.4435937 | -0.3802109 | 0.4594498 | 0.408 |
| AT(not) Sham AT(not) | 0.3611314 | 0.1122326 | -0.4128029 | 0.4514919 | 0.7739343 | 0.4659904 | 0.097 |
| Sham AT(device) WL | 0.2089765 | 0.2829288 | 0.2659251 | 0.6151687 | -0.0569486 | 0.6758793 | 0.933 |
| Sham AT(not) WL | 0.537404 | 0.1818165 | 0.0968394 | 0.2125837 | 0.4405646 | 0.2805292 | 0.116 |

AT(device), verum acupuncture in sham device-controlled trials; AT(not), verum acupuncture in sham-controlled trials without a sham device; Sham AT(device), sham device control; Sham AT(not), nondevice sham acupuncture; WL, waitlist.

Supplement 5. Results of testing inconsistency at the local level through the node splitting method: Physical function

| Side | Direct comparison | | Indirect comparison | | Difference | | p-value |
| --- | --- | --- | --- | --- | --- | --- | --- |
|  | Coefficient | SE | Coefficient | SE | Coefficient | SE |  |
| AT(device) WL | -0.0026536 | 0.3323445 | -0.0273674 | 0.7163504 | 0.0247138 | 0.7888362 | 0.975 |
| AT(device) Sham AT(device) | . | . | . | . | . | . | . |
| AT(not) WL | 0.7128861 | 0.1648611 | 0.8409375 | 0.5724092 | -0.1280515 | 0.5942205 | 0.829 |
| AT(not) Sham AT(not) | 0.3043348 | 0.1445988 | -0.514697 | 0.5880896 | 0.8190318 | 0.6063119 | 0.177 |
| Sham AT(device) WL | 0.0132682 | 0.3327081 | 0.0379819 | 0.715845 | -0.0247137 | 0.788837 | 0.975 |
| Sham AT(not) WL | 0.5925377 | 0.2271575 | 0.2321555 | 0.3038191 | 0.3603822 | 0.3802317 | 0.343 |

AT(device), verum acupuncture in sham device-controlled trials; AT(not), verum acupuncture in sham-controlled trials without a sham device; Sham AT(device), sham device control; Sham AT(not), nondevice sham acupuncture; WL, waitlist.

Supplement 6. Characteristics of included studies

| **Study ID (First author, year)** | **Country** | **Sample size (analyzed)** | **Mean age  (yr)** | **(A) Acupuncture group** | | **(S) Sham group** | | **(W) Waitlist group** | **Other intervention** | **Treatment duration** | **Outcomes of interest (posttreatment value)** | **Timepoint for outcome assessment** | **Adverse events** |
| --- | --- | --- | --- | --- | --- | --- | --- | --- | --- | --- | --- | --- | --- |
|  |  |  |  | **Style** | **Protocol** | **Style** | **Protocol** |  |  |  |  |  |  |
| Chen 2013 | USA | (A) 104, (S) 109 | (A) 60.5 ± 11.1 (S) 60.4 ± 11.7 | Acupuncture (device) | Standardized acupuncture | Sham (device) | Non-penetrating Streitberger needles at acupuncture points | - | Exercise-based physical therapy  (all groups) | 12 weeks | WOMAC (pain, function, stiffness) | 12 weeks | (A) 47 cases (agitation 2, bruising 1, fatigue 1, increased pain 22, redness/infection 1, muscle soreness 6, swelling 6, weakness 1, other 7) (S) 31 cases (bruising 1, fatigue 1, increased pain 16, muscle soreness 2, swelling 5, tearfulness 1, other 5) |
| Foster 2007 | England | (A) 113, (S) 115, (W) 105 (pain) / (A) 113, (S) 110, (W) 105 (function) | (A) 63.1 ± 8.7 (S) 62.8 ± 9.4 (W) 63.8 ± 8.3 | Acupuncture (device) | Semi-standardized acupuncture | Sham (device) | Non-penetrating Streitberger needles at acupuncture points | O | Exercise-based physical therapy  (all groups) | 3 weeks | WOMAC (pain, function) | 6 weeks | (A) 5 cases (pain 1, sleepiness 1, fainting 1, nausea 1, swelling around the treated knee 1) (S) none (W) none |
| Hinman 2014 | Australia | (A) 64, (W) 69 | (A) 64.3 ± 8.6 (W) 62.7 ± 8.7 | Acupuncture (not device) | Semi-standardized acupuncture | - | - | O | - | 12 weeks | WOMAC (pain, function), 0-10 NRS (pain) | 12 weeks | (A) 9 cases (increased knee pain 5, pain in other areas 1, tingling 1, tiredness 1, swelling 1) (W) none |
| Lam 2021 | China | (A) 42, (S) 41 | (A) 62.7 ± 7.0 (S) 63.4 ± 6.7 | Acupuncture (not device) | Individualized acupuncture | Sham (not device) | Non-penetrating acupuncture at acupuncture points | - | - | 4 weeks | 0-100 mm VAS (pain) | 4 weeks | (A) 18 cases (pain in needle insertion 7, pain in needle adjustment 5, pain in needle removal 1, bleeding 1, bruise 3, numbness 1) (S) 3 cases (pain in needle insertion 1, pain in needle adjustment 2) |
| Lin 2018 | China | (A) 21, (S) 21 | (A) 59.5 ± 7.5 (S) 60.0 ± 7.3 | Acupuncture (not device) | Semi-standardized acupuncture | Sham (not device) | Shallow needling at non-acupuncture points | - | - | 8 weeks | WOMAC (pain, function), 0-100 mm VAS (pain) | 8 weeks | (A) 2 cases (needling pain after treatment 1, hematoma 1) (S) 1 case (needling pain after treatment 1) |
| Miller 2011 | Israel | (A) 28, (S) 27 | (A) 70.3 ± 10.2 (S) 72.2 ± 7.2 | Acupuncture (device) | Semi-standardized acupuncture | Sham (device) | Non-penetrating empty needle tube at acupuncture points | - | Standard therapy (e.g., NSAIDs, steroid injection)  (all groups) | 8 weeks | Knee society score (pain, function) | 8 weeks | None |
| Min 2006 | South Korea | (A) 40, (S) 38 | (A) 58.9 ± 5.6 (S) 60.0 ± 5.0 | Acupuncture (device) | Individualized acupuncture | Sham (device) | Non-penetrating Park's sham needle at acupuncture points | - | - | 4 weeks | WOMAC (pain, function, stiffness), 0-100 mm VAS (pain) | 4 weeks | NR |
| Penagos-Martinez 2021 | Spain | (A) 15, (S) 14 | (A) 62.31 ± 8.45 (S) 62.08 ± 7.71 | Acupuncture (not device) | Standardized acupuncture | Sham (not device) | Non-penetrating needle at acupuncture points | - | Therapeutic exercise  (all groups) | 4 weeks | WOMAC (pain, function, stiffness), 0-10 cm VAS (pain) | 4 weeks | None |
| Scharf 2006 | Germany | (A) 326, (S) 365, (W) 316 | (A) 62.8 ± 9.9 (S) 63.0 ± 10.1 (W) 62.6 ± 10.1 | Acupuncture (not device) | Semi-standardized acupuncture | Sham (not device) | Minimal-depth needling without stimulation at defined non-acupuncture points | O | Physical therapy  (all groups) | 6 weeks | WOMAC (pain, function, stiffness) | 13 weeks | (A) 179 cases (including arthralgia 16, bone pain 10, hematoma 12, back pain 8, joint lock 9) (S) 177 cases (including arthralgia 15, bone pain 10, hematoma 18, back pain 11, joint lock 8) (W) 159 cases (including arthralgia 13, bone pain 13, hematoma 1, back pain 6, joint lock 5) |
| Takeda 1994 | Canada | (A) 20, (S) 20 | (A) 63.00 ± 8.78 (S) 60.20 ± 9.75 | Acupuncture (not device) | Standardized acupuncture | Sham (not device) | Superficial needling at non-acupuncture points (approximately 1 inch from the acupuncture points) | - | - | 3 weeks | WOMAC (pain, function, stiffness) | 3 weeks | NR |
| Tu 2021 | China | (A) 145, (S) 146 | (A) 63.0 ± 7.2 (S) 62.8 ± 7.6 | Acupuncture (not device) | Semi-standardized acupuncture | Sham (not device) | Superficial needling at non-acupuncture points | - | - | 8 weeks | WOMAC (pain, function, stiffness), 0-10 NRS (pain) | 8 weeks | (A) 23 cases (subcutaneous hematomas 10, post-needling pain 13) (S) 19 cases (subcutaneous hematomas 9, post-needling pain 10) |
| Williamson 2007 | England | (A) 60, (W) 61 | (A) 72.4 ± 7.71 (W) 69.6 ± 10 | Acupuncture (not device) | Semi-standardized acupuncture | - | - | O | - | 6 weeks | 0-10 cm VAS (pain) | 7 weeks | No adverse responses to treatment occurred in any of the groups, beyond occasional minor bruising and bleeding in the acupuncture group. |
| Witt 2005 | Germany | (A) 145, (S) 73, (W) 67 | (A) 64.5 ± 6.4 (S) 63.4 ± 6.6 (W) 63.6 ± 6.7 | Acupuncture (not device) | Semi-standardized acupuncture | Sham (not device) | Superficial needling at non-acupuncture points | O | - | 8 weeks | WOMAC (pain, function, stiffness) | 8 weeks | (A) 24 cases (small hematoma or bleeding 18, other side-effects, such as needling pain 6) (S) 16 cases (small hematoma or bleeding 9, local inflammation at the needling site 1, other side effects 6) |
| Witt 2006 | Germany | (A) 235, (W) 228 | Not reported | Acupuncture (not device) | Individualized acupuncture | - | - | O | - | 3 months | WOMAC (pain, function, stiffness) | 3 months | NR |
| Yu 2021 | Taiwan | (A) 61, (S) 31 | (A) 64.79 ± 9.86 (S) 66.35 ± 10.56 | Acupuncture (not device) | Standardized acupuncture | Sham (not device) | Non-penetrating needle at acupuncture points in the upper abdomen (CV12, ST21) | - | - | 1 day | 0-10 cm VAS (pain) | 1 day | None |

NR, not reported; NRS, numeric rating scale; VAS, visual analogue scale; WOMAC, Western Ontario and McMaster Universities Osteoarthritis Index.

Supplement 7. Details of acupuncture treatment method

| **Study ID (First author, year)** | **Acupuncture points** | **Depth of insertion** | **Needle retention time** | **Needle size** | **De-qi** | **Treatment frequency** | **Number of treatment sessions** |
| --- | --- | --- | --- | --- | --- | --- | --- |
| Chen 2013 | GB34, SP9, ST36, ST35, EX-LE5, UB60, GB39, SP6, KI3 *If both knees had pain >3/10, both were treated. | 2-30 mm | 20 min | 1.2 inches, 8 gauge | Achieving | once or twice a week | 12 |
| Foster 2007 | SP9, SP10, ST34, ST35, ST36, EX-LE5, GB34, LI4, TH5, SP6, LR3, ST44, KI3, BI60, GB41 *no data on side | 5-25 mm | 25-35 min | 0.3 × 30 mm | Achieving | twice a week | 6 |
| Hinman 2014 | SP9, SP10, ST34, ST35, ST36, LR7, LR8, LR9, KI10, BL39, BL40, BL57, GB34, GB35, GB36, local extra points in the hamstring muscles, ST40, LR3, SP6, GB41, BL60, BL21, BL22, BL23, GB30, GB31, ear knee point, DU20, LI11, GV14, BL11 *Acupuncturists were permitted to treat both knees. | NR | 20 min | 0.25 × 40 mm | NR | once or twice a week | 8~12 |
| Lam 2021 | Acupuncture points (1–2 cm away from the point of tenderness, spasm or pain) along the meridian sinews near the knee *affected side | 10-20 mm | 30 min | 0.30 x 40 mm | NR | three times a week (first 2 weeks), twice a week (last 2 weeks) | 10 |
| Lin 2018 | ST34, ST35, ST36, EX-LE2, EX-LE5, GB33, GB34, SP9, SP10, LR8, GB31, GB36, GB39, GB41, ST40, ST41, LR3, BL60, SP6, KI3, LI4 *affected side | 10-30 mm | 20 min | 0.30 × 25 mm 0.30 × 40 mm | Achieving | 3 times a week | 24 |
| Miller 2011 | GB34, LI11, close Ah-shi point (opposite side)/SP5, EX-LE2, ST35, EX-LE5 (painful side)/shu stream point on the meridian involved with the knee pain/a local point around the knee | NR | 20 min | 0.16 mm diameter | NR | twice a week | 16 |
| Min 2006 | -Pain at upper and dorsal to the medial condyle of the tibia: HT8, SP2, LR1, SP1 -Pain at the medial end of the popliteal crease, dorsal to the medial condyle of the tibia: KI10, LR8, LU8, LR4 -Pain at ventral and distal to the head of the fibula: BL66, GB43, LI1, GB44 -Pain in the middle of the popiliteal fibula crease: LI1, BL67, ST36, BL40 -Pain at the lower edge of the patella, lateral to the patella ligament: SI5, LI5, GB41, ST43 -Pain in the medial part of the popliteal fossa between the tendons of the semitendinosus and semimembranosus muscles: LU8, KI7, SP3, ST41 *opposite side | 1-5 mm | 20 min | 0.25 × 40 mm | Achieving | twice a week | 8 |
| Penagos-Martinez 2021 | ST34, ST36, ST36, EX-LE5, GB34, SP9, SP10, LR3 *affected side | 5-30 mm | 30 sec | 0.30 x 40 mm | NR | once a week | 4 |
| Scharf 2006 | ST34, ST36, EX-LE5, SP9, SP10, GB34 *affected side *optional: 1–4 Ah-shi points (unilateral), 1–2 of 16 defined distant points (bilateral according to TCM) | 5-35 mm | 20-30 min | 0.3 mm diameter | Achieving | NR | 10 |
| Takeda 1994 | SP9, ST35, GB34, Extra 31, Extra 32 | NR | 30 min | 0.23 x 30 mm | Achieving | three times a week | 9 |
| Tu 2021 | ST35, EX-LE5, LR8, GB33, Ah-shi point *3 additional points: ST32, ST34, SP10, EX-LE2, ST36, ST40, LR3, LR7, LR9, SP6, KI3, SP4, BL39, BL57, BL40, KI10, GB31, GB34, GB36, GB39, BL60, GB41 *affected side | ≥ 10 mm | 30 min | 0.25 x 25-40 mm | Achieving | three times a week | 24 |
| Williamson 2007 | SP10, ST35, EX-LE5, ST36, SP9, GB34, LR3 | NR | 20 min | 1 inch, 0.25 gauge | Achieving | once a week | 6 |
| Witt 2005 | -at least 6 local points from the followings: ST34, ST35, ST36, SP9, SP10, BL40, KI10, GB33, GB34, LR8, EX-LE2, EX-LE5 -at least 2 distant points from the followings: SP4, SP5, SP6, ST6, BL20, BL57, BL58, BL60, BL62, KI3 | NR | 30 min | 20-40 mm length | Achieving | twice a week (first 4 weeks), once a week (last 4 weeks) | 12 |
| Witt 2006 | NR | NR | NR | NR | NR | NR | 15 |
| Yu 2021 | LI11, HT3, TE10 or GB34, SP9, EX-LE2 *bilateral | 15-30 mm | 20 min | 0.25 × 40 mm | NR | once | 1 |

NR, not reported

**Supplement 8. Risk of bias summary for all included studies**


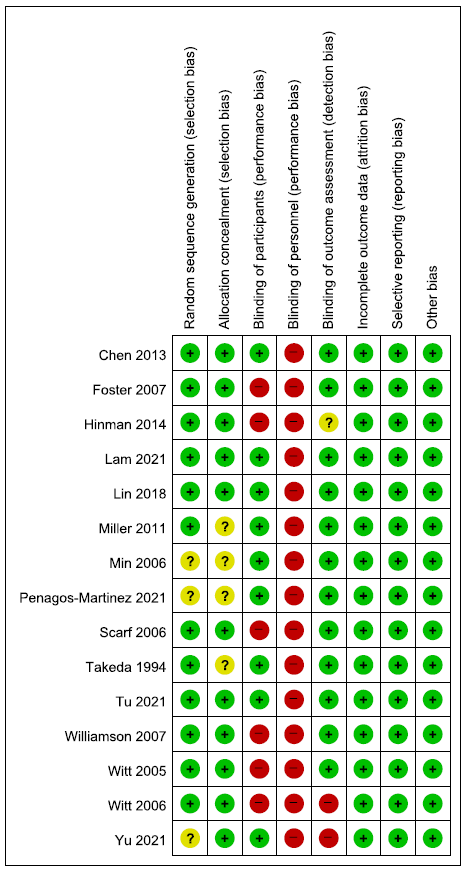
**- Risk of bias tool**

Low, unclear, and high risk, respectively, are represented with the following symbols: “+”, “?”, and “-”.

**- Revised risk of bias tool (RoB 2)**


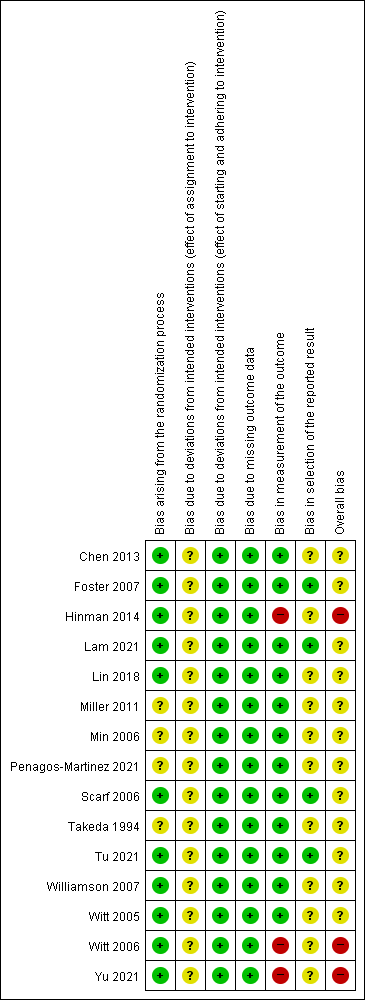


Low risk, some concerns, and high risk, respectively, are represented with the following symbols: “+”, “?”, and “-”.

**Supplement 9. Forest plot: Pain intensity**

AT(device), verum acupuncture in sham device-controlled trials; AT(not), verum acupuncture in sham-controlled trials without a sham device; Sham AT(device), sham device control; Sham AT(not), nondevice sham acupuncture; WL, waitlist.

**Supplement 10. Funnel plot: Pain intensity**

A, verum acupuncture in sham device-controlled trials; B, verum acupuncture in sham-controlled trials without a sham device; C, sham device control; D, nondevice sham acupuncture; E, waitlist.

**Supplement 11. SUCRA plot: Pain intensity**

AT(device), verum acupuncture in sham device-controlled trials; AT(not), verum acupuncture in sham-controlled trials without a sham device; Sham AT(device), sham device control; Sham AT(not), nondevice sham acupuncture; WL, waitlist.

**Supplement 12. Forest plot: Physical function**

AT(device), verum acupuncture in sham device-controlled trials; AT(not), verum acupuncture in sham-controlled trials without a sham device; Sham AT(device), sham device control; Sham AT(not), nondevice sham acupuncture; WL, waitlist.

**Supplement 13. Funnel plot: Physical function**

A, verum acupuncture in sham device-controlled trials; B, verum acupuncture in sham-controlled trials without a sham device; C, sham device control; D, nondevice sham acupuncture; E, waitlist.

**Supplement 14. SUCRA plot: Physical function**

AT(device), verum acupuncture in sham device-controlled trials; AT(not), verum acupuncture in sham-controlled trials without a sham device; Sham AT(device), sham device control; Sham AT(not), nondevice sham acupuncture; WL, waitlist.

**Supplement 15. Quality of evidence (GRADE)**

1. Pain

| Comparison | | Direct estimate | Indirect estimate | Network meta-analysis |
| --- | --- | --- | --- | --- |
| AT(device) | AT(not) | - | Moderate Risk of bias (-1) | Moderate Risk of bias (-1) |
| AT(device) | Sham AT(device) | Moderate Risk of bias (-1) | Moderate Risk of bias (-1) | Low Risk of bias (-1) Imprecision (-1) |
| AT(device) | Sham AT(not) | - | Moderate Risk of bias (-1) | Low Risk of bias (-1) Imprecision (-1) |
| AT(device) | WL | Moderate Risk of bias (-1) | Moderate Risk of bias (-1) | Low Risk of bias (-1) Imprecision (-1) |
| AT(not) | Sham AT(device) | - | Moderate Risk of bias (-1) | Low Risk of bias (-1) Imprecision (-1) |
| AT(not) | Sham AT(not) | Moderate Risk of bias (-1) | Moderate Risk of bias (-1) | Moderate Risk of bias (-1) |
| AT(not) | WL | Moderate Risk of bias (-1) | Moderate Risk of bias (-1) | Moderate Risk of bias (-1) |
| Sham AT(device) | Sham AT(not) | - | Moderate Risk of bias (-1) | Low Risk of bias (-1) Imprecision (-1) |
| Sham AT(device) | WL | Moderate Risk of bias (-1) | Moderate Risk of bias (-1) | Low Risk of bias (-1) Imprecision (-1) |
| Sham AT(not) | WL | Moderate Risk of bias (-1) | Moderate Risk of bias (-1) | Moderate Risk of bias (-1) |

1. Function

| Comparison | | Direct estimate | Indirect estimate | Network meta-analysis |
| --- | --- | --- | --- | --- |
| AT(device) | AT(not) | - | Moderate Risk of bias (-1) | Moderate Risk of bias (-1) |
| AT(device) | Sham AT(device) | Moderate Risk of bias (-1) | Moderate Risk of bias (-1) | Low Risk of bias (-1) Imprecision (-1) |
| AT(device) | Sham AT(not) | - | Moderate Risk of bias (-1) | Low Risk of bias (-1) Imprecision (-1) |
| AT(device) | WL | Moderate Risk of bias (-1) | Moderate Risk of bias (-1) | Low Risk of bias (-1) Imprecision (-1) |
| AT(not) | Sham AT(device) | - | Moderate Risk of bias (-1) | Moderate Risk of bias (-1) |
| AT(not) | Sham AT(not) | Moderate Risk of bias (-1) | Moderate Risk of bias (-1) | Low Risk of bias (-1) Imprecision (-1) |
| AT(not) | WL | Moderate Risk of bias (-1) | Moderate Risk of bias (-1) | Moderate Risk of bias (-1) |
| Sham AT(device) | Sham AT(not) | - | Moderate Risk of bias (-1) | Low Risk of bias (-1) Imprecision (-1) |
| Sham AT(device) | WL | Moderate Risk of bias (-1) | Moderate Risk of bias (-1) | Low Risk of bias (-1) Imprecision (-1) |
| Sham AT(not) | WL | Moderate Risk of bias (-1) | Moderate Risk of bias (-1) | Moderate Risk of bias (-1) |

AT(device), verum acupuncture in sham device-controlled trials; AT(not), verum acupuncture in sham-controlled trials without a sham device; Sham AT(device), sham device control; Sham AT(not), nondevice sham acupuncture; WL, waitlist.
